# Exotic plantations differ in “nursing” an understory invader: A probe into invasional meltdown

**DOI:** 10.1002/ece3.11398

**Published:** 2024-05-23

**Authors:** Tong Wang, Haifang Li, Xue Yang, Zeyu Zhang, Shengwen Liu, Jinming Yang, Huicui Lu, Shimei Li, Mingyan Li, Xiao Guo, Yuwu Li

**Affiliations:** ^1^ College of Landscape Architecture and Forestry Qingdao Agricultural University Qingdao Shandong Province China; ^2^ Academy of Dongying Efficient Agricultural Technology and Industry on Saline and Alkaline Land in Collaboration with Qingdao Agricultural University Dongying Shandong Province China

**Keywords:** functional traits, invasion mechanism, light condition, plant–plant interaction, soil microbes, soil stoichiometry, understory invader

## Abstract

Forest plantations most likely promote exotic plant invasion. Using an in situ monitoring method, this study investigated the traits correlated with growth and reproduction of an understory invader, *Phytolacca americana* L., and ecological factors including understory irradiance, soil stoichiometry and microbial patterns associated with these traits in different exotic plantations of *Robinia pseudoacacia* L. and *Pinus thunbergii* Parl. at Mount Lao, Qingdao, China. We found that the traits of *P. americana* underneath the *R. pseudoacacia* stand might be situated at the fast side of the trait economic spectrum. The *R. pseudoacacia* stand appeared to “nurse” *P. americana*. Furthermore, we intended to explain the nurse effects of *R. pseudoacacia* stands by examining their ecological factors. First, the *R. pseudoacacia* stand created understory light attenuation, which matched the sciophilous feature of *P. americana*. Second, the soil beneath the *R. pseudoacacia* stand might benefit *P. americana* more since the soil has greater resource availability. Third, a higher microbial diversity was found in the soil derived from *P. americana* underneath the *R. pseudoacacia* stand. A greater abundance of plant pathogens was detected in the soil derived from *P. americana* in the *R. pseudoacacia* stand, while more abundant mycorrhizal fungi were detected in the *P. thunbergii* stand. We speculate that plant pathogens can defend *P. americana* from aggression from other understory competitors. The mycorrhizal fungi in the *P. thunbergii* stand might benefit *P. americana* while simultaneously benefiting other understory plants. Intensive competition from other plants might interfere with *P. americana*. The potential relationships between plant performance and ecological factors may explain the invasion mechanism of *P. americana.* The present study provides a novel insight on the facilitative effects of exotic tree plantation on an exotic herb through the modification of soil biota, with implications for the biocontrol of invasive species and forest management and conservation.

## Graphical abstract

The present study explored the nurse effects of two exotic plantations, *Robinia pseudoacacia* L. and *Pinus thunbergii* Parl. on the understory invader, *Phytolacca americana* L. in Mount Lao of North China. We found that the *R. pseudoacacia* stand nursed the invasion of *P. americana* since the understory invader performed better underneath the *R. pseudoacacia* stand. We intend to explain the nurse effect using ecological factors and found that the understory irradiance, soil fertility, and microbial pattern likely influenced the performance of *P. americana*.

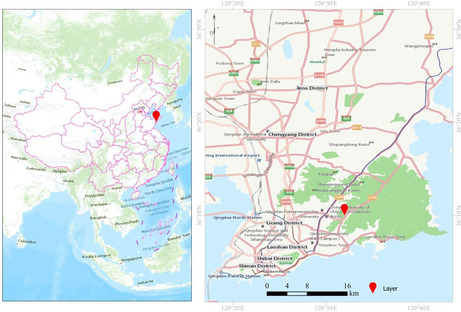

## 1 INTRODUCTION

Forests provides benign understory habitats for several shrubs and herbs, protects them from the harm of overheating, promotes their survival and establishment and causes beneficiary “nurse effects” (Cavieres et al., 2005). Therefore, an increasing number of species have invaded forest habitats, especially plantations of exotic trees (Kumar et al., 2020; Lapin et al., 2019). In addition, nurse plants can play a role in the ecological engineering of species by modifying soil conditions and “luring” the invasion of exotic plants. Invasional meltdown and mutualistic interactions among exotic invaders may underlie “nurse effects,” which may promote their coinvasion and boost the impacts of invasion on native ecosystems (Simberloff & Von Holle, 1999). For instance, Flory and Bauer (2014) reported that the invasion of *Microstegium vimineum* enhanced habitat resource availability and facilitated the secondary invasion of *Alliaria petiolata* in a long‐term field experiment. Niu et al. (2007) reported that the soil biota at sites occupied by exotic plants inhibited the growth of native species more than they did at sites occupied by invasive species. Jordan et al. (2008) showed that invasive plants modify the soil microbiota, which promotes invasion directly or via “cross‐facilitation” of other invasive species. Zhang et al. (2020) found that exotic plants were more competitive than their native counterparts in soil conditioned by other exotics and that soil microbes drove the invasion success of exotic plants in a multispecies pot experiment.

The facilitative effects of nurse plants can be evaluated by the amelioration of the growth of beneficiary species (Al‐Namazi & Bonser, 2020; Garrote et al., 2022; Lozano et al., 2020). Plant life‐history traits are representative of plant fitness since they are equivalents of growth, survival and reproduction to a certain extent (Laughlin et al., 2020). Leaf and root functional traits are common indices for the resource use of above‐ (e.g., light and carbon) and belowground resources (e.g., soil nutrients) (Funk et al., 2016). The leaf trait economic spectrum and the root trait economic spectrum have been proposed for the analysis of correlations between traits and resource use (Fajardo & Siefert, 2018; Weemstra et al., 2016). For instance, plants with a high specific leaf area (SLA) are considered to exhibit rapid growth since plants with a high SLA usually return quickly to their investment in nutrients and leaf mass and thus exhibit a high growth rate (Leishman et al., 2007; Montesinos, 2022). The foliar nutrient concentration (e.g., leaf nitrogen concentration) usually positively correlates with photosynthetic efficiency, as nitrogen is a constituent of chlorophyll and photosynthesis‐related enzymes (Taiz & Zeiger, 2010; Wang et al., 2017). In correspondence with leaf functional traits, root functional traits such as the root nutrient reserve may indicate the resource use of belowground nutrients (Weemstra et al., 2016). However, plant root systems are diverse; for example, storage roots function as repositories for nutrient reserves (Vanderschuren & Agusti, 2020). The nutrient concentration of storage roots is associated with asexual reproductive capacity and stress tolerance (Yin et al., 2020).

Afforestation with exotic plantations has been pervasive across natural, seminatural and artificial habitats all over the globe and has caused both beneficiary and harmful impacts to the local ecosystems (Baard et al., 2024; Gómez‐González et al., 2024; Reisman‐Berman et al., 2019). Mount Lao, which is located in Qingdao city, China, has a vast area of artificial tree plantations (Zhang et al., 2015). Due to the poor site conditions for plant growth (e.g., thin soil layer, granitic body, and soil nutrient deficiency), several tolerable tree species have been introduced for silviculture and revegetation. Two exotic species, *R. pseudoacacia* (originating from North America) and *Pinus thunbergii* (originating from Korean Peninsula and Japan), which were introduced in the early 20th century and commonly used for afforestation in Shandong Province, China, dominate plantations and usually form pure stands (Li et al., 2022). *Phytolacca americana* L. (originating from North America) has been officially listed as an exotic invasive species by the Ministry of Ecology and Environment of the People's Republic of China since this species can establish pervasive monostands, rapidly deplete soil nutrients, exclude native species by resource competition and allelopathy and threaten local biodiversity (Ministry of Ecology and Environment of the People's Republic of China & Chinese Academy of Sciences, 2016). Since this species has a morphology similar to that of its native congener, also a traditional Chinese medicine, *Phytolacca acinosa* Roxb., people tend to eat *P. americana* for medical use by mistake, and the poisonous chemical substances of *P. americana* induce diarrhea and cause other health problems (Xiao et al., 2022). Due to the sciophilous character of the exotic herb *P. americana*, the understory of *R. pseudoacacia* and *P. thunbergii* stands has been invaded by this species, and several birds assist in the dispersal of this species in the forest ecosystem of Mount Lao (Xiao et al., 2022). Thus, the plantations of *R. pseudoacacia* and *P. thunbergii* most likely served as nurses for *P. americana*. In particular, the *Robinia pseudoacacia* stand has been favored by several exotic plant species, and an invasive meltdown—the mutual benefit between invaders—was proven between *R. pseudoacacia* and plant invaders (Medvecká et al., 2018). In addition, using a pot experiment, Chen et al. (2019) found that *P. americana* performed better in soil collected from *R. pseudoacacia* plantations than in soil derived from natural forests due to the greater resource availability of *R. pseudoacacia* plantations. In this study, traits relevant to the growth and reproduction (indicators of invasion) of *P. americana* in the understory of *R. pseudoacacia* and *P. thunbergii* stands were compared in situ. Additionally, enlightened by previous studies, the soil beneath each sampled plant in each stand type was analyzed for its stoichiometry and microbial pattern (indicators of invasibility). Two hypotheses were proposed as follows:

1. The stand type determines the trait performance relevant to the growth and reproduction of *P. americana*, and the *R. pseudoacacia* stand had a stronger nurse effect on *P. americana* than did the *P. thunbergii* stand.
2. Ecological factors including understory irradiance, soil stoichiometric and microbial patterns, determine the trait performance of *P. americana* and are related to its invasion success in *R. pseudoacacia* stands.

The present study is important for conservation practitioners, forest managers and forestry decision‐makers since practical strategies for the management of understory invaders can be proposed based on the current outcomes.

## 2 MATERIALS AND METHODS

### 2.1 Sampling site

**FIGURE 1:**
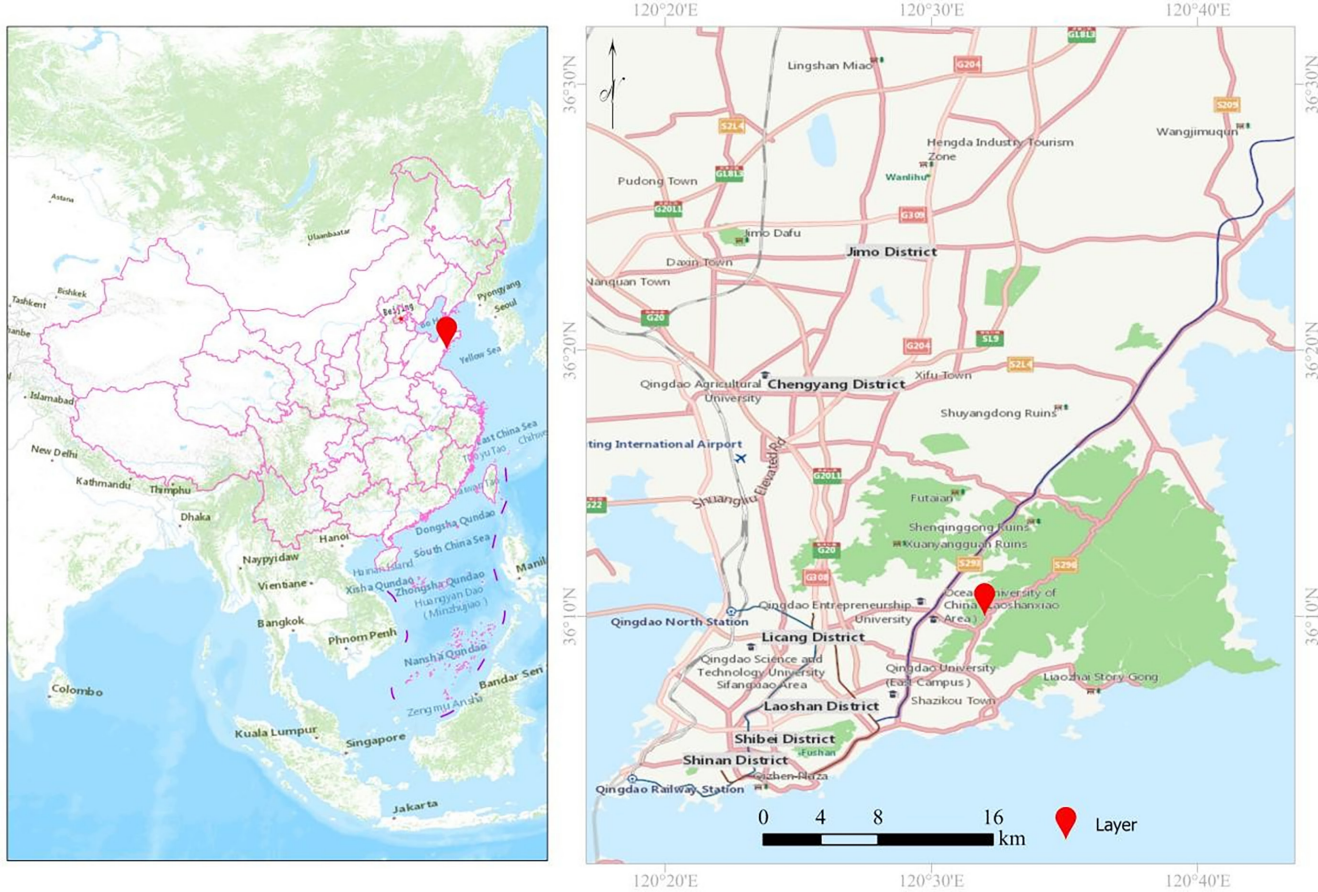
The location of sampling site for *Phytolacca americana* in the understory of respective three pure stands of *Robinia pseudoacacia* and *Pinus thunbergii*.

From 9th to 10th September 2022, *P. americana* was sampled in six forest plots (20 m × 30 m) with three pure stands of *R. pseudoacacia* and *P. thunbergii* (for detailed information, *see* Table 1) in the Nanjiushui region of Mount Lao, Qingdao, China (36°10′ N, 120°32′ E; Figure 1).

**TABLE 1:** The characteristics for the respective three sampling 20 m × 30 m plots of *Robinia pseudoacacia* and *Pinus thunbergii* pure stands.

Stand type	Abundance	Average DBH (cm, diameter at breast height)	Average height (m)	Average age (year)	Average slope (°)	Understory irradiance (lux)
*R. pseudoacacia*	44 ± 4^a^	18.31 ± 0.67^a^	8.22 ± 0.43^a^	32.94 ± 2.81^a^	29.0 ± 3.8^a^	3677.8 ± 475.8^b^
*P. thunbergii*	106 ± 35^a^	10.57 ± 0.96^b^	6.55 ± 0.02^b^	21.79 ± 1.42^b^	26.3 ± 1.5^a^	8665.0 ± 2715.1^a^

*Note*: Values represent mean ± standard error. Different lowercase letters indicate that the values are significantly different at *p* < .05.

Qingdao is located in the temperate region of China and has a hybrid monsoon climate and marine climate. The average annual temperature is 12.7°C, and the average annual precipitation is 700 ± 100 mm (Guo et al., 2023). The sampled plants were similar in size within the understory of each tree stand. The plant height was recorded. The distance between any two sample plants was greater than 5 m. Five, 6, and 6 *P. americana* plants (for a total of 17 plants) were sampled from the 3 *R. pseudoacacia plots*, and 6, 7 and 7 *P. americana* plants (for a total of 20 plants) were sampled from the 3 *P. thunbergii* plots. Five leaves at the third node of the branches were randomly harvested and labeled. A piece of storage roots (diameter size: 1–4 cm) – morphologically and physiologically modified underground organs with a major function of nutrient reserve were cut from the belowground parts of each sampled plant and labeled. The soil underneath the plant (around the roots, 20 cm in depth) was sampled after the sampling of leaves and storage roots. All the sampled materials were immediately placed in an ice box after each sampling and returned to the refrigerator at −20°C for storage in the laboratory.

### 2.2 Functional trait measurement

High‐definition images of the five sampled leaves (600 dpi) were generated using an Epson Perfection v19 scanner with a ruler on the scanning board. The area per leaf was calculated using ImageJ 1.46 (National Institutes of Health, Bethesda, Maryland, USA). Then, each leaf was labeled and oven‐dried at 72°C for 48 h. The dry weight per leaf was determined using an analytical balance. The specific leaf area (SLA) was calculated as follows:

SLA=dryweightperleaf/leaf areaperleaf

The sampled storage roots were oven‐dried at 72°C for 48 h. Then, the dry materials of the leaves and storage roots were ground into powder for the analyses of carbon (C) and nitrogen (N) concentrations per leaf mass (leaf C and leaf N) and per root mass (root C and root N) (EuroEA3000 CHNS‐O analyzer, Euro Vector, Italy). The leaf carbon/nitrogen ratio (leaf C/N) and root carbon/nitrogen ratio (root C/N) were calculated as follows:

leafC/N=leafC/leafN

rootC/N=rootC/rootN

The remnant plant materials were oven‐dried at 72°C for 72 h. Then, the biomass of each sampled plant was determined.

### 2.3 Soil elemental analysis

The soil in Mount Lao (brown soil) is an Eutric Cambisol according to the soil classification system of the United Nations (FAO & IIASA, 2023). The sampled soil was placed in a dry and ventilated room and naturally weathered to a constant weight. Then, each soil sample was ground into powder and sieved. The soil carbon and nitrogen contents (soil C and soil N) were measured using a EuroEA3000 CHNS‐O analyzer (EuroVector, Italy) (Wang et al., 2017). Then, the soil C/N was calculated as the soil C/soil N.

### 2.4 Sequencing for soil bacteria and fungi

#### 2.4.1 Extraction of genomic DNA

The CTAB method was used to extract whole‐genome DNA from the soil samples. The DNA concentration and purity were monitored on 1% agarose gels. Then, sterilized water was added to dilute the DNA to 1 ng μL^−1^.

#### 2.4.2 Amplicon generation

Specific primers (e.g., 16S V4: 515F806R, 18S V4: 528F‐706R, 18S V9: 1380F‐1510R, etc.) were used to amplify the 16S rRNA/18SrRNA/ITS genes of distinct regions (16S V4/16S V3/16S V3‐V4/16S V4‐V5, 18S V4/18S V9, ITS1/ITS2, and Arc V4) with barcodes. All PCRs were performed with 15 μL of Phusion® High‐Fidelity PCR Master Mix (New England Biolabs), 2 μM forward and reverse primers, and approximately 10 ng of template DNA. Thermal cycling included 1 min of initial denaturation at 98°C, 30 cycles of denaturation at 98°C for 10 s, 30 s of annealing at 50°C, 30 s of elongation at 72°C and a 5 min extension at 72°C.

#### 2.4.3 PCR product quantification and qualification

The same volumes of 1× loading buffer (containing SYB green) and PCR products were mixed, and electrophoresis was performed on a 2% agarose gel for detection. The PCR products were mixed at equal density ratios. The Qiagen Gel Extraction Kit was subsequently used to purify the mixture of PCR products (Qiagen, Germany).

#### 2.4.4 Library preparation and sequencing

A TruSeq® DNA PCR‐Free Sample Preparation Kit (Illumina, USA) was used to generate sequencing libraries. Library quality was assessed using a Qubit@ 2.0 Fluorometer (Thermo Scientific) and an Agilent Bioanalyzer 2100 system. Finally, an Illumina NovaSeq platform was used for sequencing the library, and 250 bp paired‐end reads were produced.

### 2.5 Sequencing data processing

#### 2.5.1 Paired‐end read assembly and quality control

##### Data split

The unique barcodes of paired‐end reads were assigned to the samples, which were subsequently truncated by removing the barcode and primer sequence.

##### Sequence assembly

FLASH (V1.2.7, http://ccb.jhu.edu/software/FLASH/) was used to merge the paired‐end reads.

##### Data filtration

Quality filtering of the raw tags was performed under specific filtering conditions. Then, high‐quality clean tags were acquired through the QIIME (V1.9.1, http://qiime.org/scripts/split_libraries_fastq.html) quality control process.

##### Chimera removal

The UCHIME algorithm (UCHIME Algorithm, http://www.drive5.com/usearch/manual/uchime_algo.html) was used for comparisons between the tags and the reference database (Silva database). Then, the chimeric sequences were detected and removed. The effective tags were obtained.

#### 2.5.2 OTU clustering and species annotation

##### OTU production

Uparse software (Uparse v7.0.1001, http://drive5.com/uparse/) was used for sequence analysis. Sequences with more than 97% similarity were categorized into the same OTUs. A representative sequence of each OTU was screened for further annotation.

##### Species annotation

The Mothur algorithm was used to annotate taxonomy according to the Silva Database (http://www.arb‐silva.de/).

##### Data normalization

A standard sequence number corresponding to the sample with the least number of sequences was used to normalize the abundance of OTUs.

##### Alpha diversity

Shannon's index was used for the analysis of species diversity within a sample. The index in our samples were calculated with QIIME (version 1.9.1) and visualized using R software (version 2.15.3).

##### Beta diversity

Beta diversity (β‐diversity), which indicates the complexity of the community composition, was determined through weighted and unweighted UniFrac distances in QIIME (version 1.9.1).

### 2.6 Data analysis

Independent sample *t* tests were used to detect differences in growth traits, including biomass and SLA. leaf N, leaf C/N, root N, and root C/N; soil stoichiometry, including soil N and soil C/N; and indices for soil microbial diversity, including observed species and Shannon's index derived from *P. americana* in the understory of different forest stands (SPSS 22.0; IBM, NY, USA). Nonmetric multidimensional scaling (NMDS) was used to analyze the difference in soil microbial β diversity derived from *P. americana* in the understory of different forest stands (R software, version 2.15.3). Redundancy analysis (RDA) was performed to evaluate the correlation between the growth traits of *P. americana* and the soil properties (Canoco 5, Microcomputer Power, NY, USA).

## 3 RESULTS

### 3.1 Growth traits

**FIGURE 2:**
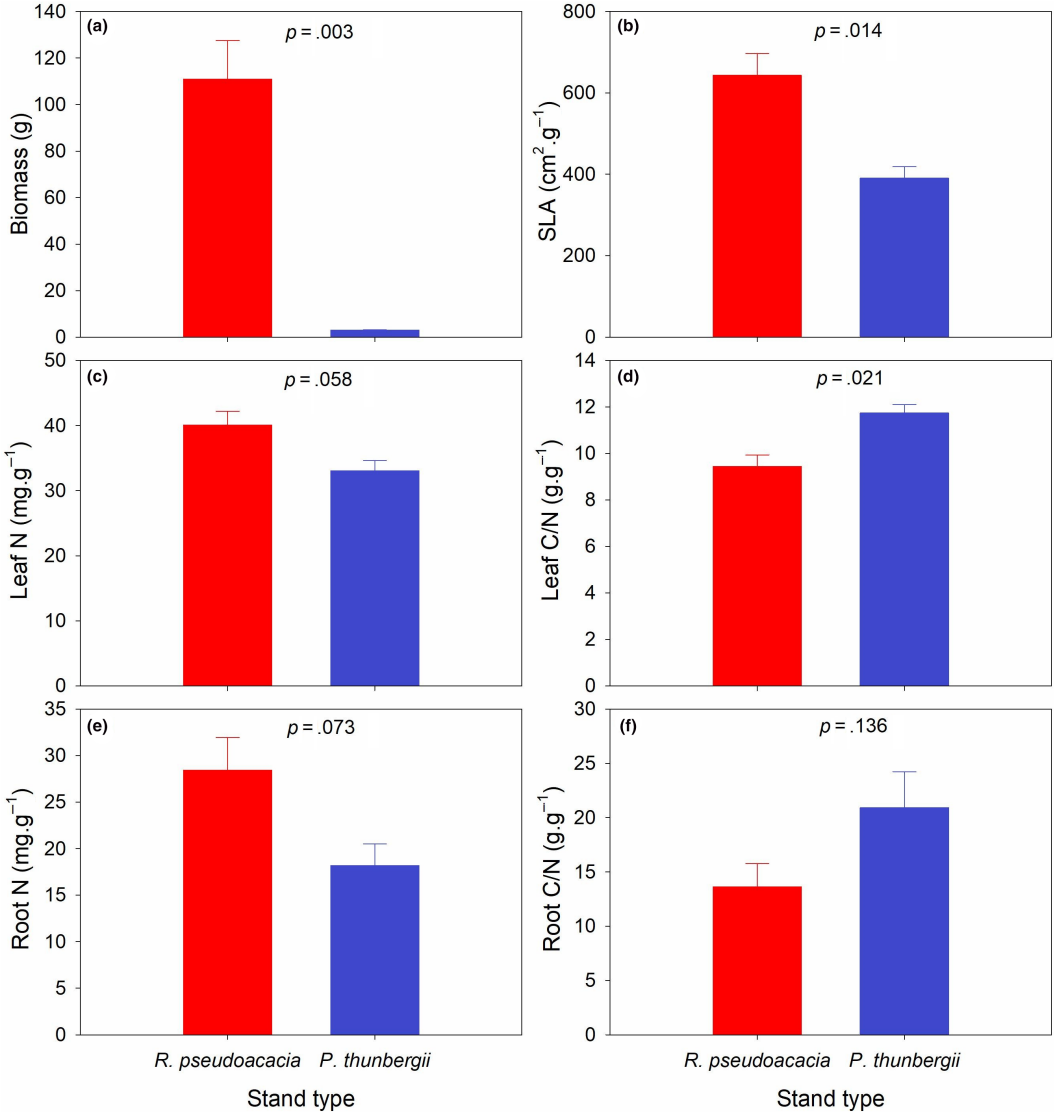
Comparison in the growth traits including (a) biomass, (b) SLA, (c) leaf N, (d) leaf C/N, (e) root N and (f) root C/N of *Phytolacca americana* underneath different stand types of *Robinia pseudoacacia* and *Pinus thunbergii*. Values are mean ± standard error.

The biomass, SLA, leaf N, and root N of *P. americana* were significantly greater underneath the *R. pseudoacacia* stand than underneath the *P. thunbergii* stand (*p* < .1) (Figure 2a–c,e). The leaf C/N of *P. americana* were significantly lower underneath the *R. pseudoacacia* stand than underneath the *P. thunbergii* stand (*p* < .1), while no significant difference of root C/N was shown for *P. americana* underneath different stands (*p* > .1) (Figure 2d,f).

### 3.2 Soil stoichiometry

**FIGURE 3:**
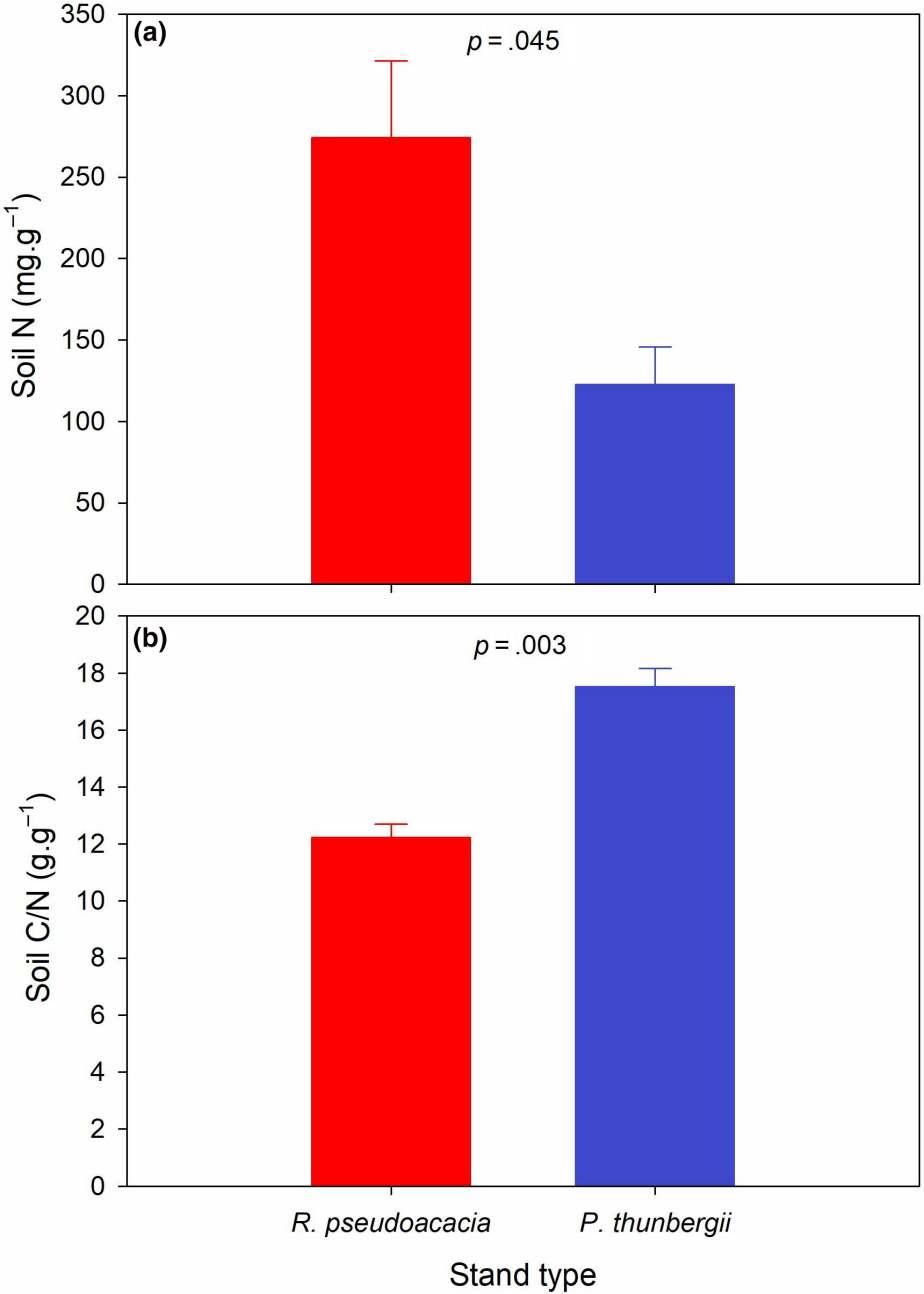
Comparison in the soil stoichiometry including: (a) soil N and (b) soil C/N derived from *Phytolacca americana* underneath different stand types of *Robinia pseudoacacia* and *Pinus thunbergii*. Values are mean ± standard error.

A significantly greater soil N and a significantly lower soil C/N were shown in the soil derived from *P. americana* underneath the *R. pseudoacacia* stand than in that derived from the *P. thunbergii* stand (*p* < .1) (Figure 3a,b).

### 3.3 Soil microbes

#### 3.3.1 Diversity

**FIGURE 4:**
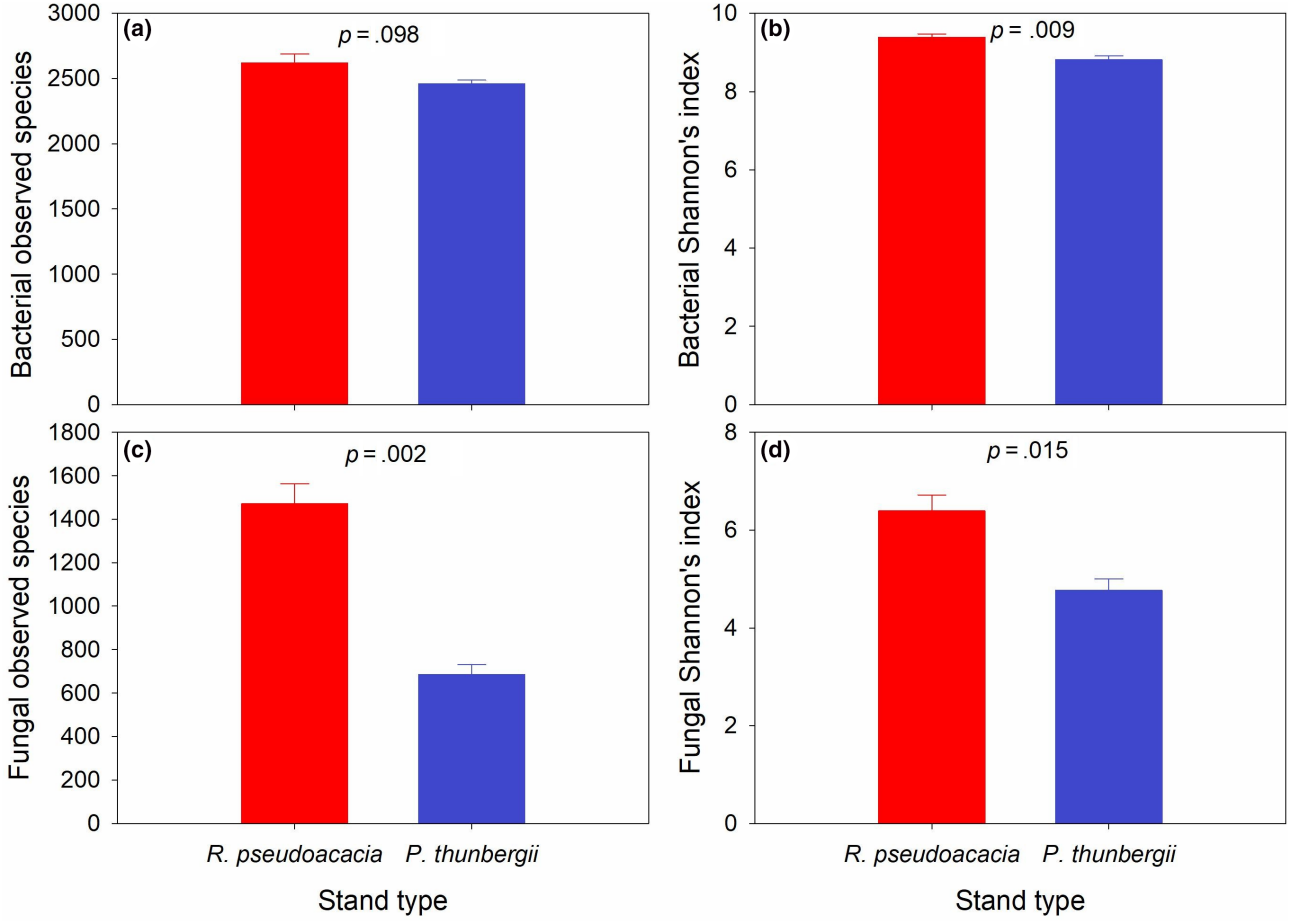
Comparison in the soil microbial diversity including: (a) bacterial observed species, (b) bacterial Shannon's index, (c) fungal observed species and (d) fungal Shannon's index derived from *Phytolacca americana* underneath different stand types of *Robinia pseudoacacia* and *Pinus thunbergii*. Values are mean ± standard error.

Generally, the observed species and Shannon's index of bacteria and fungi were significantly greater for the soil derived from *P. americana* underneath the *R. pseudoacacia* stand than for that derived from the soil underneath the *P. thunbergii* stand (*p* < .1) (Figure 4a–d).

#### 3.3.2 Generic composition

**FIGURE 5:**
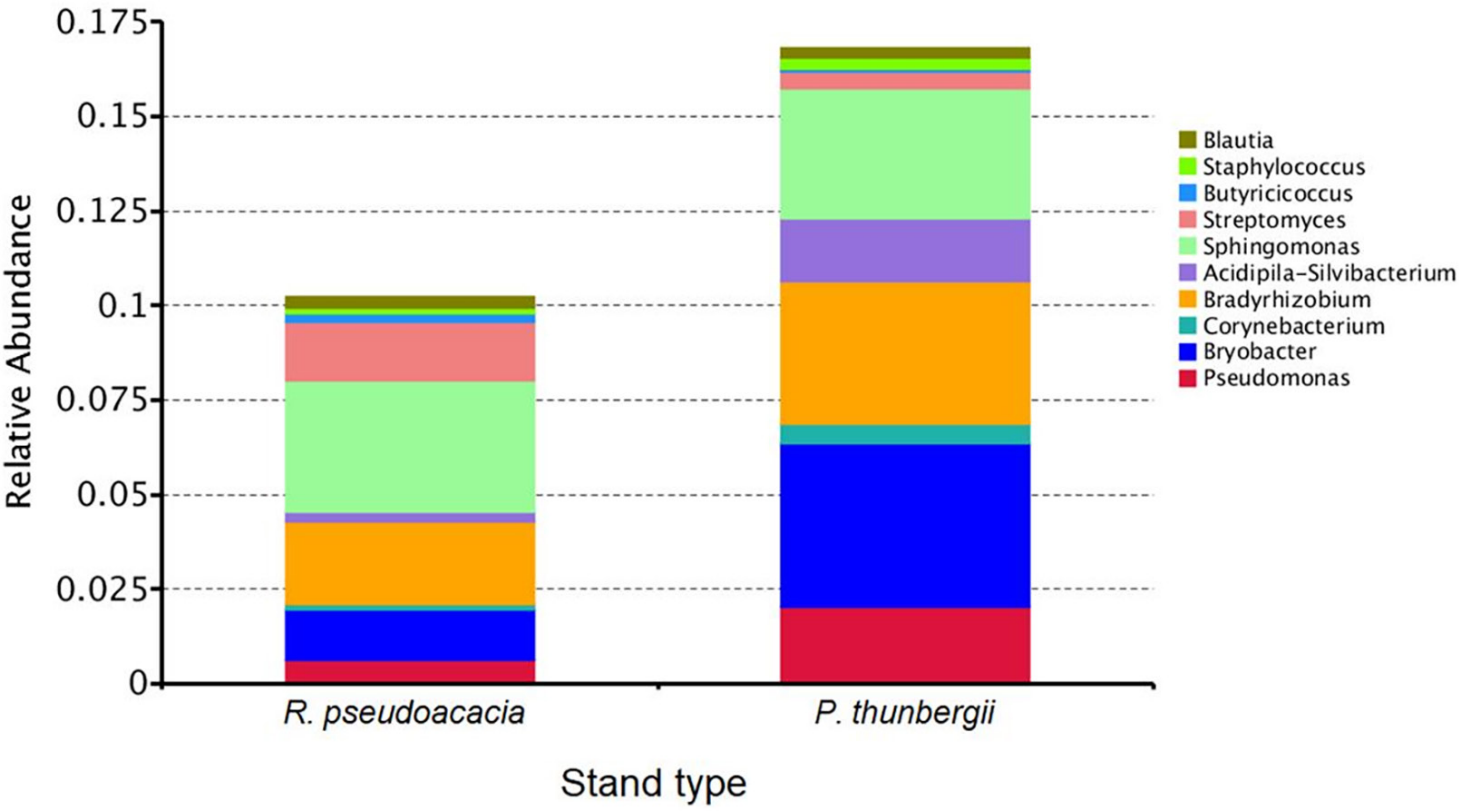
Comparison in the relative abundance of top 10 soil bacterial genera derived from *Phytolacca americana* underneath different stand types of *Robinia pseudoacacia* and *Pinus thunbergii*.

Considering the soil bacteria, *P. americana* accumulated a greater relative abundance of *Streptomyces* but a lower relative abundance of *Acidipila‐Silvibacterium*, *Bradyrhizobium*, *Corynebacterium*, *Bryobacter*, and *Pseudomonas* underneath the *R. pseudoacacia* stand than underneath the *P. thunbergii* stand (Figure 5).

**FIGURE 6:**
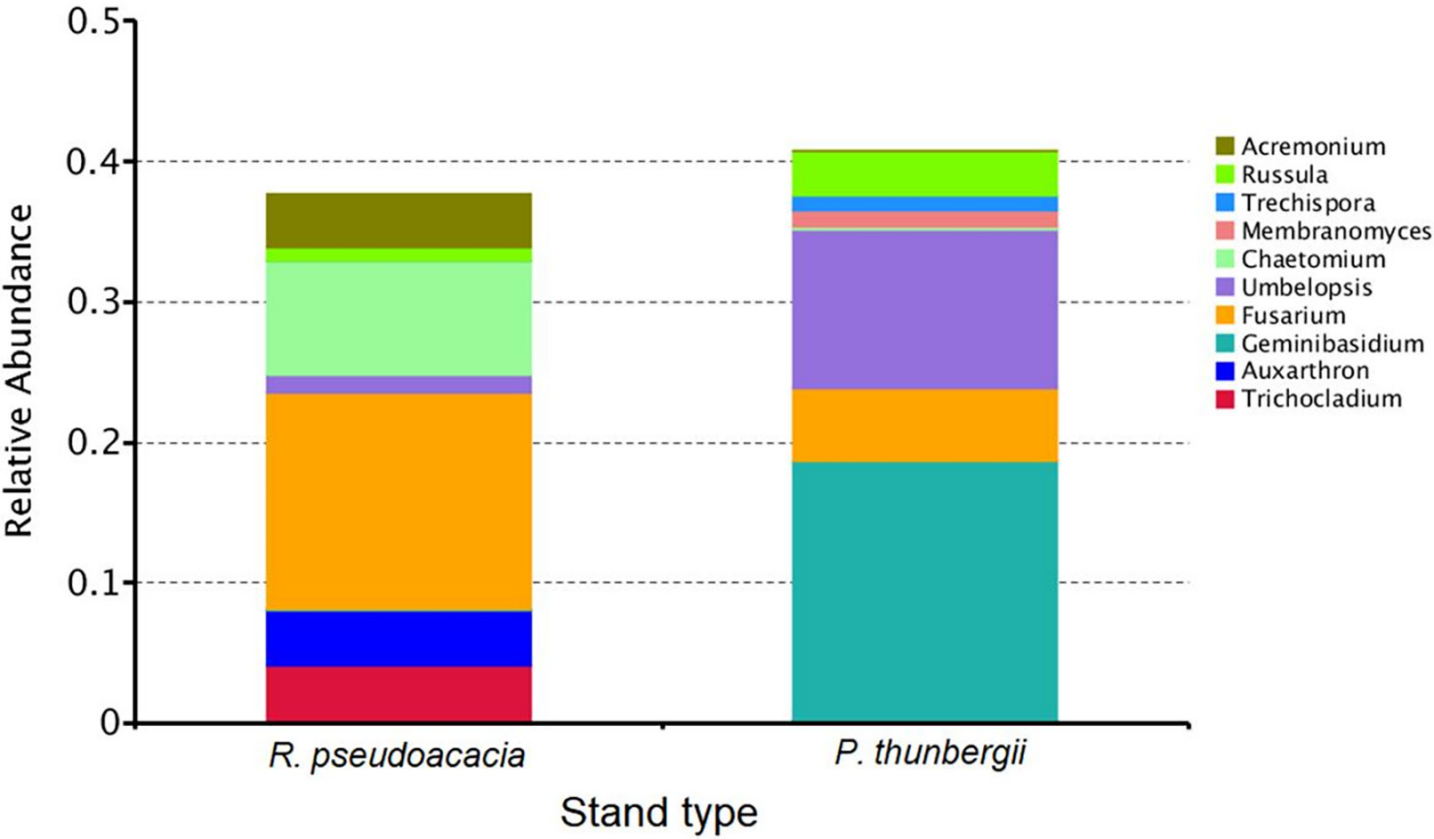
Comparison in the relative abundance of top 10 soil fungal genera derived from *Phytolacca americana* underneath different stand types of *Robinia pseudoacacia* and *Pinus thunbergii*.

Considering soil fungi, the soil derived from *P. americana* in the understory of the *R. pseudoacacia* stand had a greater relative abundance of *Fusarium* and *Auxarthron* species than that in the understory of the *P. thunbergii* stand did, while *Russula*, *Umbelopsis*, and *Geminibasidium* species were more abundant underneath the *P. thunbergii* stand (Figure 6).

### 3.4 Correlations between plant traits and ecological factors

**FIGURE 7:**
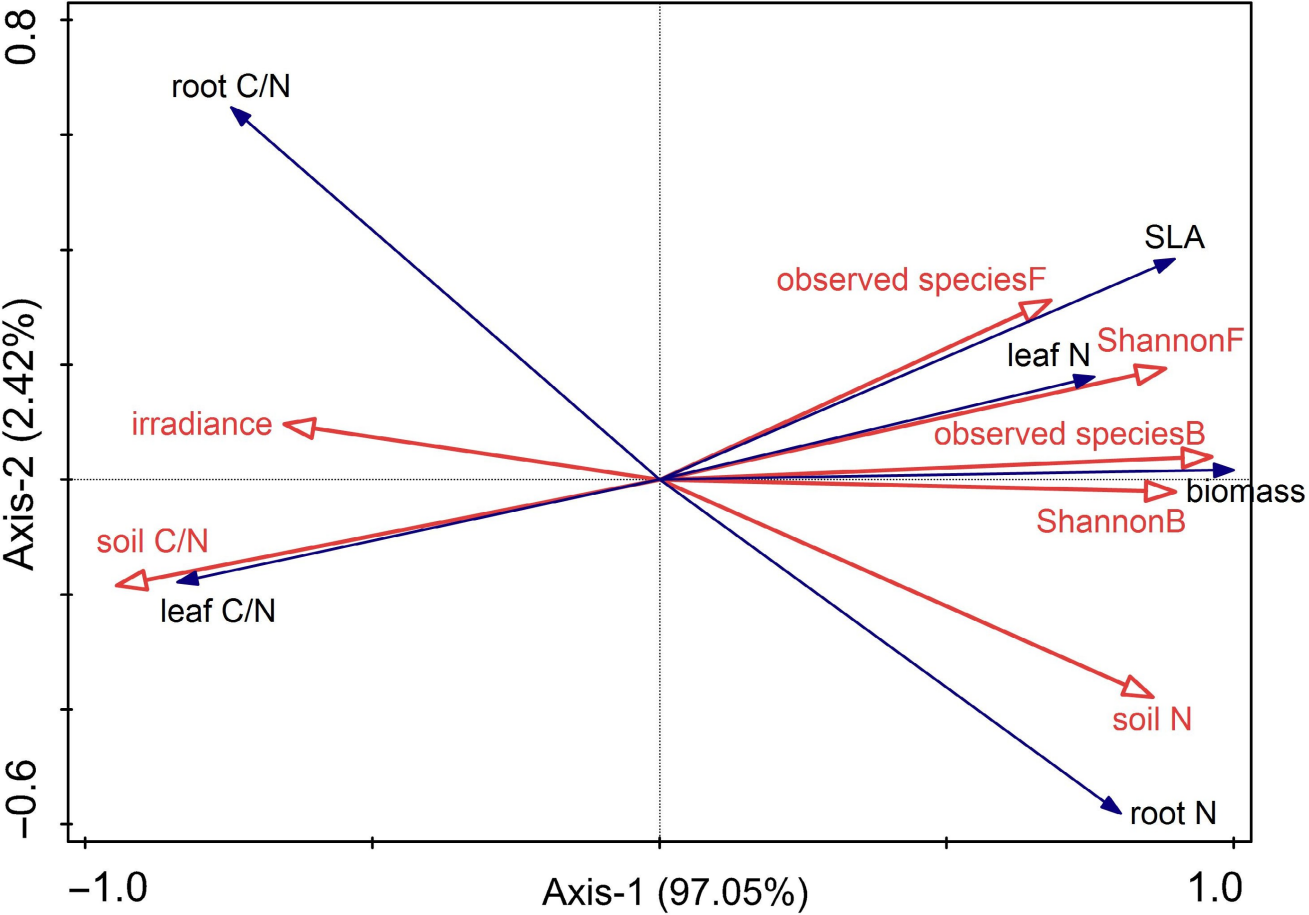
Redundancy analysis (RDA) of the correlation between growth traits of *Phytolacca americana* including biomass, SLA, leaf N, leaf C/N, root N, and root C/N, and environmental variables including understory irradiance (irradiance), soil N, soil C/N, observed species B (bacterial observed species), Shannon B (bacterial Shannon's index), observed species F (fungal observed species) and Shannon F (fungal Shannon's index).

Positive correlations were shown between biomass and bacterial observed species, biomass and bacterial Shannon's index, SLA and fungal observed species, leaf N and fungal Shannon's index, leaf C/N and soil C/N, root N and soil N (Figure 7). Negative correlations were displayed between biomass and understory irradiance, biomass and soil C/N, SLA and soil C/N, leaf N and soil C/N, leaf C/N and fungal Shannon's index, root C/N and soil N (Figure 7).

## 4 DISCUSSION

### 4.1 Advantages in terms of growth traits contributed to the thriving of invasive *P. americana* in the understory of *R. pseudoacacia* stands

An overwhelming advantage in growth was shown for *P. americana* in the understory of *R. pseudoacacia* compared to that in the understory of *P. thunbergii*. First, the biomass was approximately 36 times greater in the understory of *R. pseudoacacia* than in the understory of *P. thunbergii*. Since biomass are usually positively correlated with fecundity, biomass has been considered to represent fitness (Younginger et al., 2017). Second, *P. americana* in the understory of *R. pseudoacacia* had a greater SLA. A greater SLA usually predicts a greater relative growth rate (Leishman et al., 2007; Montesinos, 2022). Third, considering tissue stoichiometry, *P. americana* underneath the *R. pseudoacacia* stand had greater values in leaf N and root N but lower leaf C/N. The leaf trait economic spectrum indicates a positive correlation between leaf N and SLA, and a negative correlation between leaf C/N and SLA has been observed (Leishman et al., 2007). Considering the probable positive correlation between SLA and relative growth rate, a greater leaf N and a lower leaf C/N usually indicate a faster plant growth (Leishman et al., 2007; Montesinos, 2022). Root form, including root morphology, architecture and chemical parameters, is linked with root function (Iversen, 2014). On a geometric basis, roots with a herringbone branching structure have been shown to have greater nutrient absorption capacity than those with a dichotomous branching structure (Fitter et al., 1991). Thicker roots with longer lifespans and higher mycorrhizal colonization rates usually indicate higher construction costs and lower nutrient assimilation rates, while a higher root branching intensity is usually equivalent to greater proliferation in patches with abundant nutrients (Kong et al., 2014). Root morphological traits such as specific root length (SRL) and specific root area (SRA) are proxies for nutrient assimilation in plants (Weemstra et al., 2016). Root type is another deterministic factor in root function. Considering the chemical properties of roots, root N has potential positive correlations with nutrient assimilation for absorbing roots and with nutrient storage for storage roots separately, while nutrient assimilation determines the plant growth and root nutrient storage affects the clonal reproduction (Weemstra et al., 2016; Yang et al., 2020). Therefore, considering the mixing of both root types of *P. americana*, *P. americana* in the understory of *R. pseudoacacia* may have faster growth and regrowth. In summary, compared with the *P. thunbergii* stand, the *R. pseudoacacia* stand can facilitate the invasion success of *P. americana* as a nurse plant. An invasional meltdown between *R. pseudoacacia* and *P. americana* may occur, followed by a probable rigid vertical community structure of trees and herbs, which may efficiently resist the intrusion of other species and accelerate the impact of coinvasion on local ecosystems (Simberloff & Von Holle, 1999).

### 4.2 Understory irradiance, soil stoichiometry, and microbial pattern shaped the performance of *P. americana* in different exotic stands

Understory irradiance, soil stoichiometry and soil microbial patterns determine the invasion success of *P. americana*. First, a negative correlation between biomass and understory irradiance was shown, which indicates the sciophilous characteristic of *P. americana* (Xiao et al., 2022). The *R. pseudoacacia* stand can cause greater light degradation than the *P. thunbergii* stand in its understory and promotes the growth of *P. americana*. Second, in the perspective of soil stoichiometry, a higher fertility was detected in the soil in the understory of *R. pseudoacacia*, which totally synchronizes with the fast trait economics of *P. americana* (Chen et al., 2019). Redundancy analysis (RDA) revealed negative correlations between biomass and soil C/N, SLA and soil C/N, leaf N and soil C/N, root C/N and soil N; however, positive correlations between leaf C/N and soil C/N, root N and soil N were observed. Hence, greater habitat nutrient (e.g. nitrogen) availability tends to “shape” a faster trait economics for *P. americana* and results in faster growth of the exotic invader (Fajardo & Siefert, 2018; Montesinos, 2022; Weemstra et al., 2016). This observation is consistent with the fluctuating resource hypothesis on invasion mechanisms, as an increase in environmental resources provides surplus niches for invaders to colonize (Liu et al., 2020; Pearson et al., 2018; Wohlgemuth et al., 2022). Third, the observed species and Shannon's index of both bacteria and fungi increased in the soil derived from the understory of the *R. pseudoacacia* stand, which indicates the “prosperity” of the soil microbes underneath the *R. pseudoacacia* stand. Previous studies have proposed a probable correlation between soil microbial diversity especially the abundance of beneficial microbes, and invasion success (Dawson & Schrama, 2016; Meng et al., 2024). Plants and soil microbes generally mutually affect each other, as strong plant growth usually induces the proliferation of soil microbes and vice versa (Thakur et al., 2021). We also observed positive correlations between biomass and bacterial observed species, biomass and bacterial Shannon's index, SLA and fungal Shannon's index, leaf N and fungal Shannon's index.

Initial invaders may promote the secondary invasion of subsequent species by facilitating mutualism (Meng et al., 2024; Zhang et al., 2020), gathering pathogenic fungi (Inderjit & van der Putten, 2010), and destroying the native mycorrhizal system and facilitating mutualistic integration (Hale et al., 2011; Stinson et al., 2006). Considering the soil bacteria, *P. americana* “gathered” a greater abundance of *Streptomyces* in the understory of *R. pseudoacacia* stand. Several *Streptomyces* species can symbiose with plants and secrete antibiotics which may probably protect *P. americana* from infection and indirectly promote its growth (Seipke et al., 2012). However, some *Streptomyces* species, such as *S. scabies*, *S. turgidiscabies* and *S. acidiscabies*, can cause potato scab disease in tuber plants, and *P. americana*, which has a large root tuber, may be a sufferer (Bignell et al., 2010). As the ratio of “beneficial” to “harmful” *Streptomyces* species was not quantified in the present study, the effects of *Streptomyces* species on the performance of *P. americana* were uncertain. Simultaneously, greater abundances of *Bradyrhizobium* and *Bryobacter* existed in the soil derived from *P. americana* underneath the *P. thunbergii* stand. The *Bradyrhizobium* species can symbiose with legume and nonleguminous plants and assist in the assimilation of soil nutrients, such as nitrogen fixation (de Alencar Menezes Júnior et al., 2019). *Bryobacter* species aid in the decomposition of lignin and cellulose, promote the carbon cycling and are considered to benefit plant growth (Yu et al., 2016). However, since the soil fertility was lower and the diversity of neighboring species of *P. americana* was greater (based on our field observations) underneath the *P. thunbergii* stand, the beneficial effects of *Bradyrhizobium* and *Bryobacter* on *P. americana* may have weakened due to the resource limitations. Considering soil fungi, a greater proportion of mycorrhizal fungi, such as *Russula* and *Umbelopsis*, were found in the soil derived from *P. americana* in the understory of the *P. thunbergii* stand (Taniguchi et al., 2007). It is possible that the mycorrhizal fungi can improve the nutrient availability of the soil in the understory of *P. thunbergii*. However, the nitrogen concentration was significantly lower and the carbon/nitrogen ratio was significantly greater in the soil derived from *P. americana* underneath the *P. thunbergii* stands than in the soil derived from the *R. pseudoacacia* stands, which indicates a lower resource availability underneath the *P. thunbergii* stand. Mycorrhizal fungi are considered to enhance the nutrient assimilation of plants at nutrient‐poor sites (Liu et al., 2020). However, based on our observations, the understory plants underneath the *P. thunbergii* stand were more diverse. The network of mycorrhizal fungi may “assist” other plants in more than *P. americana*. As a consequence, *P. americana* in the understory of the *P. thunbergii* stands performed poorly compared to that in the understory of the *R. pseudoacacia* stands. We also found that *P. americana* accumulated a greater proportion of *Fusarium* – a common plant pathogenic fungal species in the understory of the *R. pseudoacacia* stands. According to Mangla and Callaway (2008), the invasive plant *Chromolaena odorata* contained a high abundance of soil generalist pathogenic fungi that impede the growth of native competitors. Since *Fusarium* species have been proven to be harmful to rice, wheat and other gramineous species, the accumulation of *Fusarium* in the belowground of *R. pseudoacacia* stand may inhibit the colonization and growth of native understory gramineous species such as *Miscanthus* spp. and *Stipa* spp., and thus protect the exotic *P. americana* from the competition from these native competitors (Aoki et al., 2014).

## 5 CONCLUSION

Based on the trait perspective, the *R. pseudoacacia* stands can “nurse” *P. americana* relative to the *P. thunbergii* stands. Invasional meltdown likely occurred between the exotic tree, *R. pseudoacacia* and the exotic invasive herb *P. americana*, which may exacerbate the coinvading effects on the forest ecosystem on Mount Lao. The nursing effects of the *R. pseudoacacia* stands on *P. americana* can be partly attributed to its lower understory irradiance, greater soil nutrient availability, greater soil microbial diversity and specific soil microbial structure. Considering the above findings, the application of *R. pseudoacacia* in the afforestation of mountainous areas in North China should be prudent in the context of biocontrol of exotic invasive species and forest management. The selective removal of present *R. pseudoacacia* stands seems applicable for the biocontrol of *P. americana* however remains challenging due to their profuse root resprouting ability.

However, several hypotheses need to be tested in the future studies. First, the effects of soil derived from different forest stands on understory native species need to be evaluated. In particular, since *Fusarium* species likely inhibit the colonization of native gramineous species, studies on their plant–soil feedback are urgently needed. Second, since understory irradiance, soil fertility and soil biota influence the invasiveness of *P. americana*, to discriminate the key factor in determining the invasion success of *P. americana* is worthy of investigation, which needs the assistance of manipulative experiments. Third, since both *R. pseudoacacia* and *P. americana* have long co‐existed in North America, whether the nurse effects of *R. pseudoacacia* on *P. americana* are nurtured in their introduced range is fascinating within an evolutionary context. Transcontinental collaborations are needed to explore this ecological puzzle.

## AUTHOR CONTRIBUTIONS

**Tong Wang:** Conceptualization (lead); data curation (lead); formal analysis (lead); funding acquisition (lead); methodology (lead); resources (lead); visualization (lead); writing – original draft (lead); writing – review and editing (lead). **Haifang Li:** Funding acquisition (equal); writing – review and editing (equal). **Xue Yang:** Investigation (equal); writing – original draft (supporting). **Zeyu Zhang:** Investigation (equal); writing – original draft (supporting). **Shengwen Liu:** Investigation (equal); writing – original draft (supporting). **Jinming Yang:** Formal analysis (equal); methodology (equal); visualization (equal); writing – review and editing (equal). **Huicui Lu:** Formal analysis (equal); methodology (equal); visualization (equal); writing – review and editing (equal). **Shimei Li:** Funding acquisition (equal); writing – review and editing (equal). **Mingyan Li:** Writing – review and editing (equal). **Xiao Guo:** Funding acquisition (equal); writing – review and editing (equal). **Yuwu Li:** Funding acquisition (equal); writing – review and editing (equal).

## CONFLICT OF INTEREST STATEMENT

The authors declare that they have no known competing financial interests or personal relationships that could have appeared to influence the work reported in this paper.

## Supporting information


Figures S1 and S2

## Data Availability

Dataset of *Phytolacca americana* L. traits and ecological factors is available at: Dryad https://doi.org/10.5061/dryad.n2z34tn2q.
