# Supplementary material for: Exotic plantations differ in “nursing” an understory invader: A probe into invasional meltdown
Source: Ecol Evol. 2024 May 23;14(5):e11398. doi: 10.1002/ece3.11398 (PMC11116753; doi:10.1002/ece3.11398)
Supplement: Supplementary file 1 — Figures S1 and S2 [file ECE3-14-e11398-s001.pdf]

Supplementary material

The β-diversity of the soil bacteria and fungi derived from *P. americana* underneath the *R. pseudoacacia* stand showed a significant discrepancy from that underneath the *P. thunbergii* stand (Fig. S1 and S2).

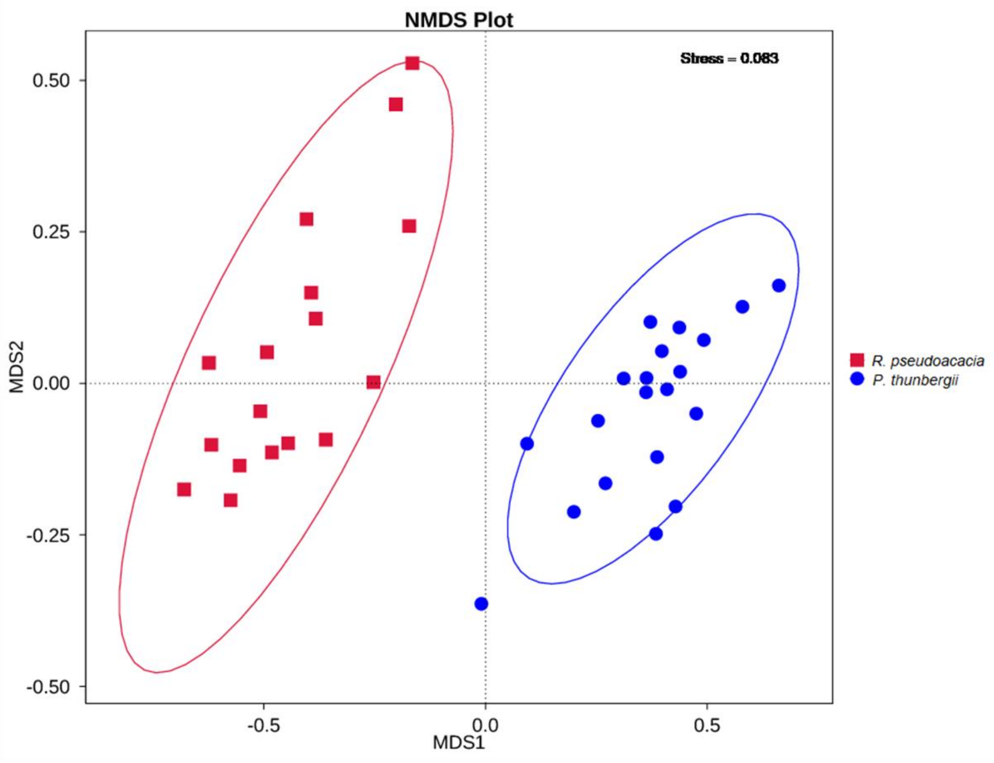

Fig. S1 Nonmetric multidimensional scaling (NMDS) plot of the soil bacterial β-diversity derived from *P. americana* underneath different stand types of *R. pseudoacacia* and *P. thunbergii*

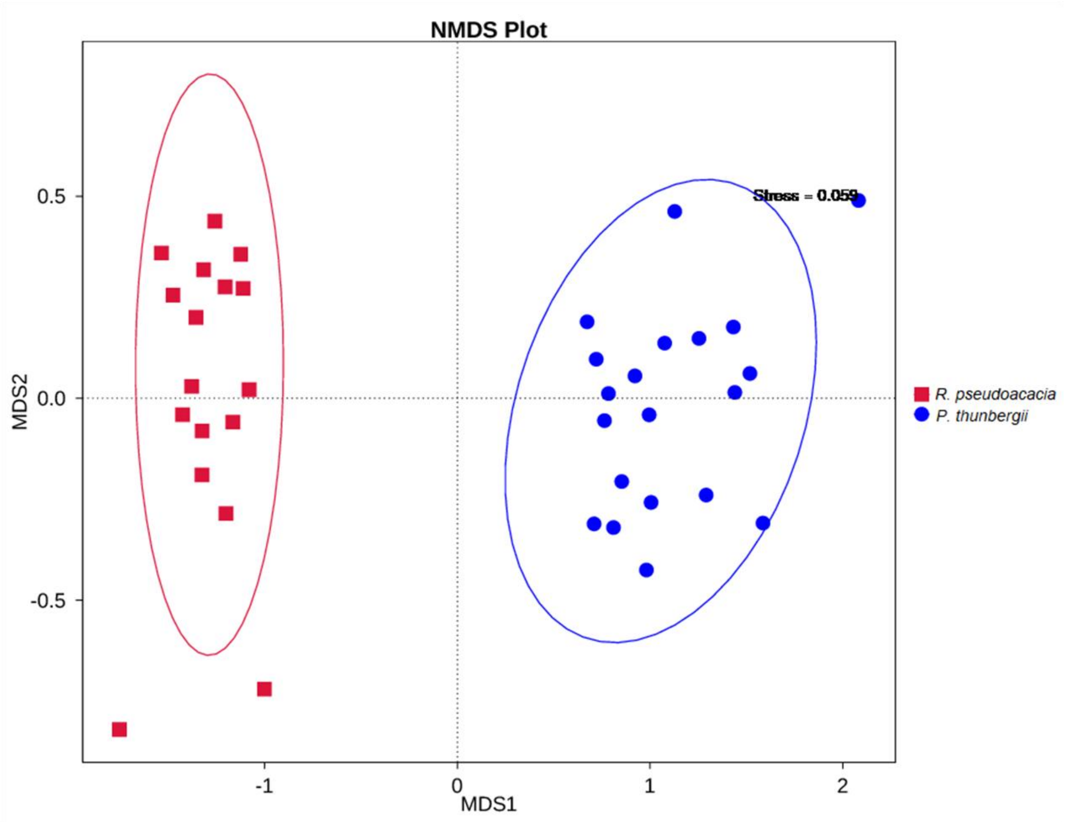

Fig. S2 Nonmetric multidimensional scaling (NMDS) plot of the soil fungal β-diversity derived from *P. americana* underneath different stand types of *R. pseudoacacia* and *P. thunbergii*
